# Simple to Complex: The Role of Actin and Microtubules in Mitochondrial Dynamics in Amoeba, Yeast, and Mammalian Cells

**DOI:** 10.3390/ijms23169402

**Published:** 2022-08-20

**Authors:** Meghan D. Jones, Kari Naylor

**Affiliations:** Department of Biology, University of Central Arkansas, 201 Donaghey Ave., Conway, AR 72035, USA

**Keywords:** actin, microtubules, mitochondrial dynamics, fission, fusion, motility, mitochondria

## Abstract

Mitochondria are complex organelles that provide energy for the cell in the form of adenosine triphosphate (ATP) and have very specific structures. For most organisms, this is a reticular or tubular mitochondrial network, while others have singular oval-shaped organelles. Nonetheless, maintenance of this structure is dependent on the mitochondrial dynamics, fission, fusion, and motility. Recently, studies have shown that the cytoskeleton has a significant role in the regulation of mitochondrial dynamics. In this review, we focus on microtubules and actin filaments and look at what is currently known about the cytoskeleton’s role in mitochondrial dynamics in complex models like mammals and yeast, as well as what is known in the simple model system, *Dictyostelium discoideum*. Understanding how the cytoskeleton is involved in mitochondrial dynamics increases our understanding of mitochondrial disease, especially neurodegenerative diseases. Increases in fission, loss of fusion, and fragmented mitochondria are seen in several neurodegenerative diseases such as Parkinson’s, Alzheimer’s, and Huntington’s disease. There is no known cure for these diseases, but new therapeutic strategies using drugs to alter mitochondrial fusion and fission activity are being considered. The future of these therapeutic studies is dependent on an in-depth understanding of the mechanisms of mitochondrial dynamics. Understanding the cytoskeleton’s role in dynamics in multiple model organisms will further our understanding of these mechanisms and could potentially uncover new therapeutic targets for these neurodegenerative diseases.

## 1. Introduction

**Figure 1:**
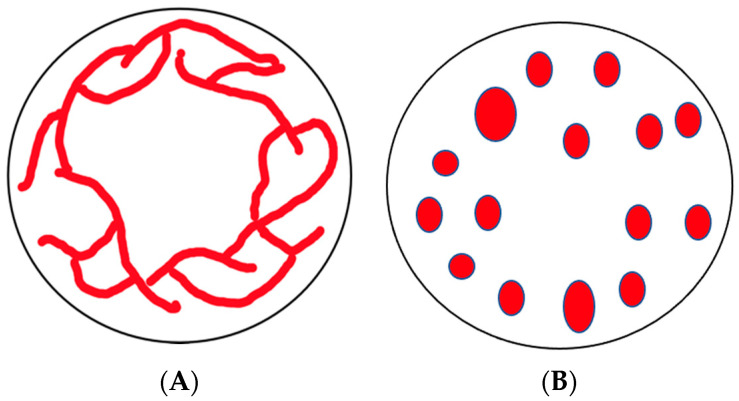
Drawing of mitochondrial networks seen in different types of cells. (**A**) The dynamic reticular network of tubular mitochondria seen in most yeast (budding and fission) and mammalian cells. (**B**) Network of individual mitochondria seen in some organisms, such as *D. discoideum*.

Mitochondria are complex organelles that provide energy for the cell in the form of adenosine triphosphate (ATP) and play an important role in many other cellular processes such as apoptosis, calcium homeostasis, and cellular differentiation [1,2,3,4]. In order to carry out these functions, the mitochondria must regulate their structure [5,6]. For most organisms, this means maintaining a reticular or tubular mitochondrial network (Figure 1) [6,7]. In other organisms, mitochondria exist as singular oval-shaped organelles (Figure 1) [8,9,10,11]. Nonetheless, maintenance of this structure is dependent on the mitochondrial dynamics, fission, fusion, and motility [12,13]. Fission refers to the division of one mitochondrion into two, and fusion refers to two mitochondria mixing their contents and becoming one [14]. Fission and fusion are balanced events [10,12,15], though there are specific cellular conditions that induce one process over the other. For example, increases in fission and mitochondrial fragmentation are induced by cellular stress, aging, and changes in metabolic state, such as high levels of glucose [16,17,18]. Fission increases are also seen with increased levels of Reactive Oxygen Species (ROS) [19], though in some organisms, such as the social amoeba *Dictyostelium discoideum*, the form of ROS determines the effect on both fission and fusion rates [20]. Fusion increases in response to cellular starvation in an effort to increase mitochondrial respiration and to mediate mitochondrial DNA damage [21,22,23,24]. It is clear that mitochondrial function and structure are intertwined and that both function and structure regulate cellular processes such as migration, cell division, embryogenesis, and disease [25]. To add to this complexity, these same cellular processes also regulate mitochondria structure and thus function [25].

Mitochondrial dynamics are essential cellular processes, and the mechanisms of these dynamics have been studied in organisms with both a reticular network, such as yeast and mammals, as well as in organisms whose mitochondria exist as individual organelles, such as the amoeba, *D. discoideum*. Recently, studies have shown that the cytoskeleton has a significant role in the regulation of mitochondrial fission and fusion in addition to motility [26]. The cytoskeleton is composed of microtubules, actin, and intermediate filaments, but for this review we will focus on microtubules and actin filaments. Actin filaments, often found near the leading edge of moving cells, play a major role in creating the force necessary for the cell to move and change its shape [27]. Microtubules are more rigid than actin and are known to form tracks that are utilized for forming the mitotic spindle during cell division, and they are necessary for organelle transport and positioning [27]. Here, we will look at what is currently known about the cytoskeleton’s role in mitochondrial dynamics in complex models like mammals and yeast, as well as what is known in the simple model system, *D. discoideum.* It is important to also understand dynamics in this simple organism, as it serves as an easy model to study neurodegenerative diseases [28]. Many neurodegenerative diseases, such as Alzheimer’s, Huntington’s, and Parkinson’s, are characterized by dysfunctional mitochondrial dynamics [29,30,31]; therefore, we will also discuss how mitochondrial dynamics contributes to neurodegeneration.

## 2. Mitochondrial Dynamics and the Cytoskeleton

Mitochondria have an integral role in many processes, the most important being cellular respiration and production of ATP through oxidative phosphorylation [1]. Mitochondria also play a role in regulating cell death, or apoptosis and autophagy [3,32]. Along with these functions, mitochondria are responsible for maintaining cellular calcium levels and serve as the main source of ROS [2,33]. All of these processes are dependent on the mitochondria continuously maintaining its structure [5,6,34,35]. In most metabolically active cells of yeast and mammals, mitochondria are tubular shaped and maintain dynamic reticular networks, while other organisms, such as *D. discoideum*, have networks of individual mitochondria (Figure 1) [6,7].

**Figure 2:**
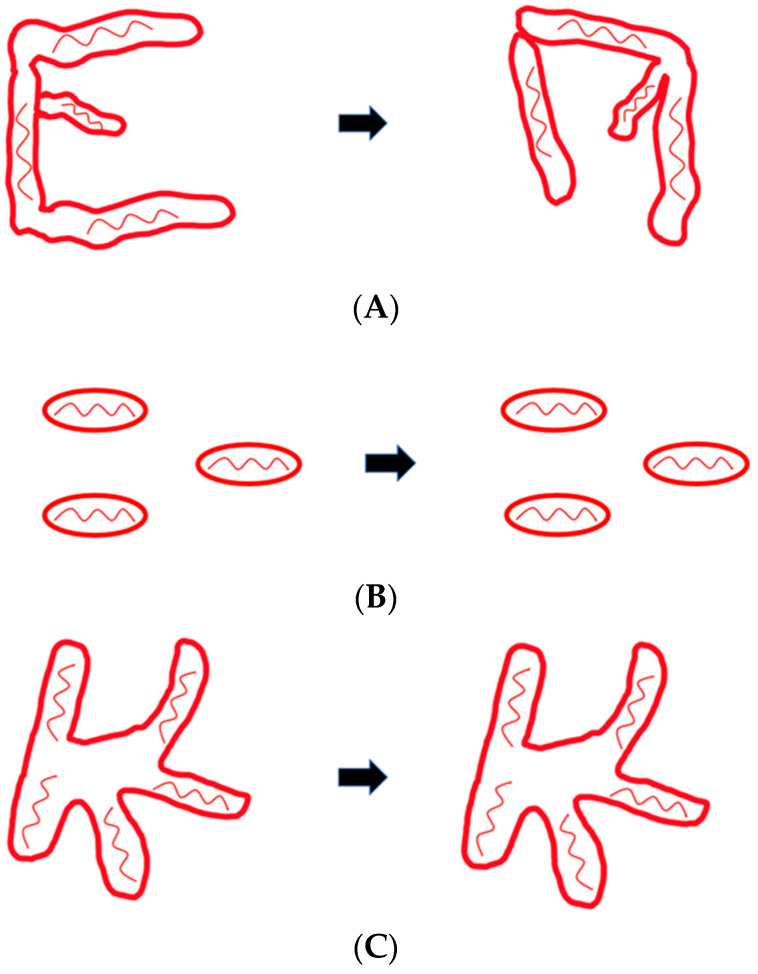
Changes in mitochondrial dynamics when fission or fusion is inhibited. (**A**) Dynamics in normal healthy cells shows how the mitochondrial network changes. (**B**) In the absence of fusion, motility is inhibited, thus, over time, mitochondrial distribution undergoes little change. (**C**) In the absence of fission, the mitochondrial network becomes too large and motility is inhibited. This again results in a similar distribution of the mitochondria over time.

Mitochondrial networks are maintained through mitochondrial dynamics, which consists of motility, fission, and fusion [12,13]. There are multiple types of mitochondrial motility seen in cells. Movements include Brownian motion, or small random passive movements, as well as directed motion, both short-range and long-range [36]. Motility is primarily necessary for mitochondria to reposition around the cell based on the cell’s energy demands [36]. Fusion, or the process of two mitochondria merging, allows for mitochondria to mix their contents and for the network to grow [37]. Fission, or the process of a mitochondrion splitting in two, allows for the network to isolate any damaged mitochondria and subsequently dispose of them through mitophagy [38]. Removing these damaged organelles regularly ensures that the network is full of only healthy mitochondria [38]. The cell also uses fission and fusion to regulate mitochondrial length [39]. Larger or longer mitochondria are more likely to go through fission, and shorter or smaller mitochondria are more likely to fuse [39]. These dynamics are connected; for example, most fusion events are followed by a fission event, rather than a subsequent fusion event, suggesting that fission and fusion occur cyclically and regulate each other [39]. When fission and fusion are not balanced, the morphology of the network changes [35]. If more fission than fusion is occurring, the network will consist of a higher number of smaller mitochondria [35]. Fusion events generally occur between one motile and one relatively stationary mitochondrion, indicating that fusion is dependent on mitochondrial motility [39]. These events require mitochondria to come into close contact with each other and can occur tip to tip or tip to a side of a tubule [39,40]. Conversely, motility is also dependent on fusion, as fusion-inhibited mammalian cells have limited mitochondrial movement, with most movements resembling Brownian motion (Figure 2B) [33]. In addition, motility is connected to mitochondrial fission [30]. Without fission, the mitochondrial network becomes too large, impeding its ability to move (Figure 2C) [41]. In conclusion, fission, fusion, and motility are interconnected, interdependent, and necessary for maintaining morphology. Defects in these dynamics lead to reduced clearance of damaged organelles and contribute to neurodegeneration [42,43].

Mitochondrial dynamics are essential to the health of the organelle and the cell. The molecular mechanisms that regulate these processes are complex but fairly well understood. Recently, it has become established that the cytoskeleton, well known for its role in organelle motility, also plays a significant role in mitochondrial fission and fusion. Much research has been carried out to determine if the cytoskeleton is directly involved in fission and fusion or if the disruption of motility indirectly alters the interconnected processes of fission and fusion. Here, we review the role of the cytoskeleton, microtubules and actin, in mitochondria motility, fission, and fusion, in neurons, other mammalian cells, yeast, and the social amoeba *D. discoideum*. We include this variety of organisms because it is clear that different mechanisms are responsible for mitochondrial dynamics in different organisms. Understanding the differences of these mechanisms helps us to understand the evolution of the machines that carry out these processes, but more importantly, using simple model systems, such as *D. discoideum*, allows us to explore dynamics without being overwhelmed by the complexity of model systems such as neurons and mammalian cells.

## 3. Microtubules and Mitochondrial Dynamics in Neurons and Mammalian Cells

The role of microtubules in mitochondrial dynamics, specifically motility, has been widely studied in mammals. Most mammalian studies on mitochondrial motility have been conducted in neurons. In mammalian neurons, mitochondria use microtubules as tracks for long-distance movement [44,45,46]. The mitochondria on these tracks have a significantly higher velocity than those moving along actin filaments [44]. For long-distance travel along axons, mitochondria move with the help of microtubule-based motors: kinesin-1, kinesin-3, and cytoplasmic dynein [46,47,48,49]. Long-distance anterograde travel (away from the cell body) is mediated by kinesin-1, as it is a highly processive motor, and its processivity can be increased by interacting with adaptor proteins. The adaptors TRAK1 and TRAK2 link microtubule motors directly to the mitochondria, or indirectly via Miro, or Mitochondrial Rho GTPase [46,50]. In cultured monkey kidney cells, defects in mitochondrial distribution are seen when the activity of Miro is inhibited [50,51]. An additional adaptor that plays a role in connecting kinesin-1 and mitochondria is syntabulin [52]. In neurons, syntabulin mediates anterograde transport of mitochondria and knockdown of this adaptor results in decreased distribution along axons [53]. Finally, loss of kinesin-3 adaptor, KIFBP (kinesin binding protein), has also been shown to alter mitochondrial distribution, though this may be by altering microtubule dynamics rather than directly affecting motility [50]. Table 1 summarizes these proteins and functions. In summary, loss of function in these adaptors that interact with microtubule motors is further support that microtubules are necessary for mitochondrial trafficking in these cells.

Along with motility, microtubules have a role in mammalian mitochondrial fission and fusion. Mitochondria must be moving to fuse, so when motility is inhibited, a decrease in fusion also occurs; this has been clearly demonstrated in mammalian neurons [54]. In mice neurons, disrupting microtubules with vincristine decreases both mitochondrial fission and fusion [55]; this may be due to a lack of motility or may be due to a more direct requirement of microtubules for the fission and fusion process. Apart from neurons, studies in other mammalian cells have linked microtubules to fission, specifically through microtubule-associated proteins (MAPs). For example, in mammalian endothelial cells, microtubule-associated tumor suppressor 1 (Mtus1) binds to mitofusins, fusion-regulating proteins on the outer mitochondrial membrane, and helps mediate fusion [56]. Knocking out Mtus1 results in decreased fusion and shorter mitochondria [56]. Studies have also suggested that the microtubule-based motor protein, kinesin-1, and the associated adaptor protein, Miro, regulate morphology, but it is unclear if this is due to their role in motility, which would indirectly affect fission and fusion [50]. Kinesin-1, along with other motor proteins, kinesin-3 and dynein, do recruit Drp1, a fission-initiating protein, to the mitochondria [50], suggesting a direct regulatory role for mitochondrial fission. A summary of the function of these proteins can be found in Table 1. While these studies suggest that microtubules are necessary for regulating mitochondrial fission and fusion in mammals, another mammalian study has shown that fusion can occur without microtubule assistance. In cultured Madin–Darby canine kidney cells (MDCK), disruption of microtubules with nocodazole does not prevent mitochondrial fusion, as measured by mitochondrial morphology [57], though it is possible that with a direct quantification assay, fusion rates (or maybe both fission and fusion) may decrease in these cells. In conclusion, microtubules do regulate motility, fission, and fusion, though many studies are still needed to determine the degree and exact method of this regulation.

## 4. Microtubules and Mitochondrial Dynamics in Yeast

Another model system used to study mitochondrial dynamics is yeast. Similar to mammals, microtubules regulate mitochondrial distribution [58]. Mitochondria appear to move along microtubule tracks by attachments at the tip or sides of growing microtubules [59]. Most mitochondrial motility in fission yeast is short range, so microtubule motors are not utilized; instead, motility is dependent on microtubule dynamics [58,60,61]. Though, one study shows that treatment of fission yeast with thiabendazole, a microtubule inhibitor, does not appear to affect mitochondrial motility [62]. Budding yeast, on the other hand, do not use microtubules for mitochondrial motility, indicating that they have a different mechanism for mitochondrial motility than fission yeast [63]. Interestingly, though it is thought that they do not use microtubules for movement, budding yeast do contain a homolog for the microtubule-based motor adaptor protein, Miro, called Gem1 [64]. Knocking out this protein results in an increase in globular mitochondria which subsequently have inheritance defects [64]. This suggests that Gem1 has a role in mitochondrial distribution, though its molecular mechanism, which is currently poorly understood, is different from that of Miro [64,65].

In fission yeast, microtubules have a clear role in mitochondrial fission. When microtubules are associated with mitochondria, the yeast fission regulatory protein, dynamin-related protein (Dnm1), is prohibited from interacting with the mitochondria; thus, interactions between mitochondria and microtubules effectively block fission [66]. Knocking out the microtubule-mitochondria binding protein, Mmb1, prohibits the microtubules from physically associating with the mitochondria and results in uncontrolled fission [66]. Destabilization of microtubules with thiabendazole induces Dnm1-dependent mitochondrial fragmentation [62]. Therefore, microtubules in fission yeast appear to downregulate fission, ensuring that a reticular mitochondrial structure is maintained. A role for microtubules for fusion in fission yeast, nor in mitochondrial fission or fusion in budding yeast, has not been established. In summary, microtubules are involved in mitochondrial motility and fission in both mammals and fission yeast. A role for microtubules in fusion, though, has only been established in mammals (Table 2).

## 5. Actin and Mitochondrial Dynamics in Mammals

In mammals, along with microtubules, actin also has an important role in mitochondrial motility. In neurons, when microtubules are scarce, mitochondria will use bundles of F-actin, known as actin cables, to travel short distances [44]. The mitochondria moving along actin cables travel at a slower velocity than those on microtubules [44]. It is unclear if these mitochondria travel using actin-based motors such as myosin [79]. Though these studies suggest that actin may assist microtubules in motility, other recent studies in neurons show that actin attachment to the mitochondria is necessary for mitochondrial anchoring [67,68]. Destabilization of actin results in a small increase in the number of moving mitochondria, suggesting that actin attachment might actually play a role in inhibiting mitochondrial motility [67]. Outside of neurons, in mice embryonic fibroblasts, Miro functions to recruit myosin motors (Myo19) to the mitochondria, allowing for actin motor-based mitochondria transport [69].

In mammals, an extensive amount of research has provided evidence for actin’s vital role in mammalian fission. Most mammalian fission is initiated at endoplasmic reticulum (ER) contact sites on the mitochondria [70]. Fission that is initiated at these sites is dependent on actin polymerization by the actin nucleator, inverted formin 2 (INF2), localized on the ER [71,72]. Activation of INF2 is in response to increased calcium levels [80], which simultaneously stops mitochondrial motility [81,82]. Following actin polymerization, actin will recruit myosin, to begin mitochondrial constriction [83]. Recent work by Yang and Svitkina show that neither myosin nor actin are forming a constriction ring, but instead myosin likely induces tension on the actin network at sites of future fission events, squeezing the mitochondria and promoting constriction [84]. After the actin-myosin complex begins the constriction, actin also has a role in recruiting dynamin-related protein-1 (Drp1) to the mitochondria to initiate fission [73]. Disruptions in the levels of F-actin result in a decrease in the amount of Drp1 that is recruited to the mitochondria, and thus decreases fission [73]. Live-cell imaging has shown that F-actin will cyclically bind to different subpopulations of mitochondria in the cell to regulate fission [72]. While actin is associated with the mitochondria, fusion is prohibited [72]. Once actin disassociates, fusion is then allowed to balance fission and recover the morphology of the mitochondrial network [72]. This indicates that actin has a role in regulating mammalian fusion in addition to fission. Additionally, other actin regulatory proteins, apart from IFN2, such as Arp2/3, cortactin, and cofilin also affect mitochondrial fission [61]. Knocking out these proteins results in decreased fission and an abundance of elongated mitochondria [61,81]. The mechanism behind how Arp2/3 and cortactin affect fission is currently poorly understood, though it is clear that branched actin regulators are involved, and they interact with Drp1 [61]. A bit more is known about cofilin, an actin destabilizer. It is involved in some forms of mitochondrial-dependent apoptosis, which requires mitochondrial fission to take place [85] and deletion of cofilin increases recruitment of Drp1 and fragmentation of mitochondria [86]. In conclusion, while it is clear in mammalian cells that actin plays a role in mitochondrial motility and is essential for many fission events, there is little known about the relationship between actin and mitochondrial fusion (Table 2).

## 6. Actin and Mitochondrial Dynamics in Yeast

**Figure 3:**
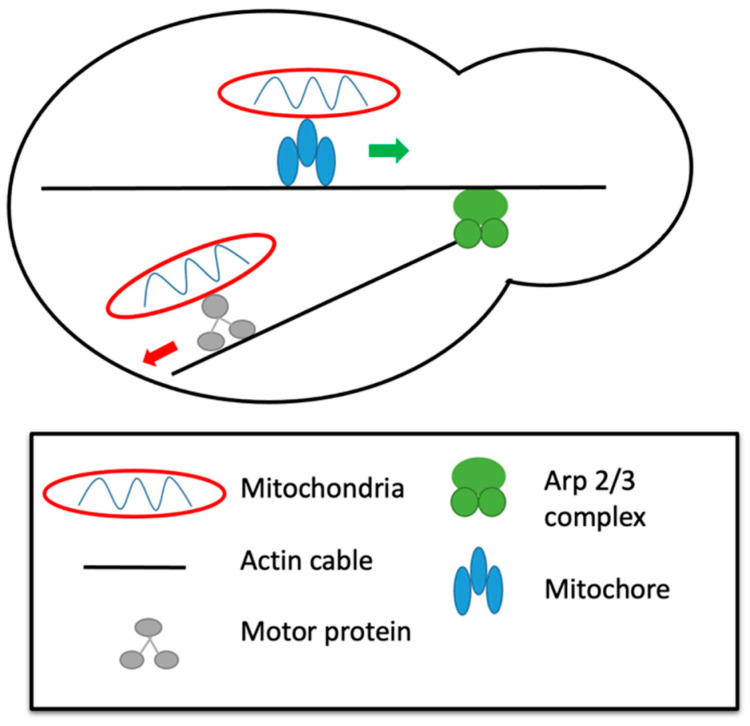
Model of two potential mechanisms of motility in budding yeast. It is thought that anterograde moving mitochondria travel along actin by attaching via the mitochore (**top**). It is also thought that motor proteins may be the point of attachment for mitochondria to actin filaments (**bottom**).

In budding yeast, studies demonstrate that actin is essential for mitochondrial motility. Live-cell imaging shows mitochondria using actin cables as tracks during movement [74,75], similar to microtubules in mammalian cells or fission yeast. The mitochondria will bind to actin cables for anterograde movement, or movement from the mother cell to daughter bud tips, as well as for retrograde movement, or movement from the daughter bud towards the mother cell (Figure 3) [63,74,75]. Destabilizing these actin cables results in loss of mitochondrial motility [75]. It is unclear if actin-based motors are used to move mitochondria along actin tracks in budding yeast, some studies clearly suggest they are not used [76], but others suggest they are [65]. It is clear that the majority of movement is generated from actin nucleation by Arp 2/3 complexes localized on the mitochondria (Figure 3) [76,77]. Following nucleation, Arp 2/3 will bind to existing actin filaments and create a branched network that is used for movement [76]. Mutations in Arp 2/3 subunits results in decreased mitochondrial movement [75]. Therefore, motility in budding yeast is dependent on actin dynamics [77]. In fact, retrograde transport of the mitochondria appears to be dependent on actin dynamics only, as retrograde mitochondrial velocity is equivalent to the retrograde disassembly of actin filaments [52]. Anterograde mitochondrial motility is dependent upon interactions with the mitochore, a protein complex made up of Mmm1, Mdm10, and Mdm12 which mediates interactions with actin filaments (Figure 3) [52].

Little is known about actin’s role in fission and fusion in yeast, whether this is because it has no role or because it has not been studied is unclear. Recently, a protein, Srv2 (suppressor of ras val-2), was identified as an inducer of fission. In budding yeast, this regulator of actin assembly interacts with Dnm1 at the mitochondria [78]. Deletion of this protein results in loss of fission due to the loss of the actin network [78]. In summary, actin regulates mitochondrial motility in both mammals and budding yeast, and it has a vital role in mammalian and most likely budding yeast fission. The role of actin in mitochondrial fusion is not known. Table 2 provides a summary of the cytoskeleton’s involvement in mitochondrial dynamics in these model systems.

## 7. *Dictyostelium discoideum* as a Model

A role for the cytoskeleton in the mitochondrial dynamics of yeast and mammals has been established, but our lab studies dynamics in the lower eukaryote, *D. discoideum*. *D. discoideum* is a eukaryotic soil-dwelling amoeba that has a unique lifestyle consisting of both unicellular and multicellular stages, making it an ideal model to study numerous diseases and signaling pathways, including mitochondrial diseases [87]. The haploid genome of *D. discoideum* was the first amoebozoan genome that was fully sequenced and is readily available on a public domain [88,89,90]. Most *D. discoideum* cells have oval-shaped mitochondria, contrary to the tubular branched mitochondria seen in other models [9,10]. Previous work in our lab has shown that *D. discoideum* carry out balanced fission and fusion to maintain mitochondrial morphology, similar to the previously studied models [10], but this process does not use the dynamin related proteins (Drp1 or dnm1) as used by yeast and mammalian cells [10,91,92].

**Figure 4:**
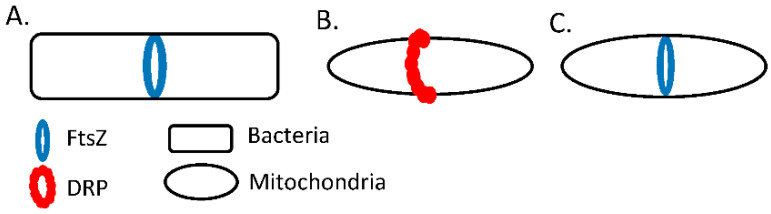
Schematic of (**A**) prokaryotic cell division, (**B**) mitochondrial fission in yeast or mammals, and (**C**) hypothesized mechanism of mitochondrial fission in *D. discoideum*. FtsZ is the primary component found in the cytokinetic ring of prokaryotic cells. DRP represents the dynamin-related proteins that mediate mitochondrial division in yeast and mammals but not *D. discoideum*.

*D. discoideum* is thought to be the link between single-cell organisms and multicellular organisms [93]. In support of this, these organisms express the prokaryotic cell division machinery, FtsZ proteins, which have been lost in many other higher eukaryotes (Figure 4) [9,94]. Work by Gilson et al. demonstrates that the FtsZs encoded by *D. discoideum* may be the master regulators of fission and fusion, suggesting that *D. discoideum* mitochondria may be more evolutionarily related to prokaryotes than to other eukaryotes like yeast (Figure 4) [9]. Mitochondria evolved from an internalized ancestor prokaryote [95], thus is it logical that as mitochondria evolved their dynamics would have as well. It is possible that *D. discoideum* mitochondrial fission is more similar to prokaryotic cell division and that as organisms evolved their mitochondria evolved different mechanisms to mediate mitochondrial dynamics. Therefore, understanding how dynamics works in *D. discoideum* will allow us to learn how mitochondrial dynamics evolved from prokaryotes to eukaryotes and provide insight into an alternative mechanism of mitochondrial dynamics.

## 8. The Cytoskeleton and Mitochondrial Dynamics in *Dictyostelium discoideum*

While we do not know as much about the cytoskeleton’s involvement in *D. discoideum* as we do in other models like mammals and yeast, a functional role in dynamics has been established. Microtubules do regulate mitochondrial motility. Rapid movement both toward and away from the microtubule organizing center (MTOC), near the nucleus, has been seen, suggesting that the mitochondria might track along microtubules [96]. Velocity of the mitochondria is significantly decreased in strains with altered microtubules, implying that microtubules are necessary for quick travel in *D. discoideum* [97,98]. Interestingly, Vlahou et al. suggest that *D. discoideum* do not use their ortholog to Miro, GemA, for mitochondrial attachment to motor proteins [99]. Kinesin-3 has been identified as the predominant plus-end orientated motor in *D. discoideum* [97], while dynein has been shown to move cargo towards either the plus or minus ends of microtubules [100], thus it is logical that these motors will prove to be required for mitochondrial motility in *D. discoideum*.

Few have studied actin’s role in motility in *D. discoideum*. Our previous work found that inhibiting actin results in fewer motile mitochondria, suggesting that they may play a role in mitochondrial motility [98]. In addition *D. discoideum* contain 13 different myosins, and a few of these have been implicated in organelle motility [101,102].

Currently, the role the cytoskeleton plays in *D. discoideum* fission and fusion is unknown. Previous work in our lab has shown that microtubules are required for fission and fusion and inhibiting them significantly reduces their rates [98]. We also deduced that actin has a smaller role in fission and fusion compared to microtubules, as disruption of actin only slows fission and fusion [98]. Microtubules having a significant role in *D. discoideum* fission is contrary to the mechanism established in mammals, which is reliant on actin [72,73]. This again supports the fact that *D. discoideum* uses an alternative mechanism for mitochondrial dynamics than budding yeast or mammalian cells.

## 9. The Cytoskeleton and Mitochondrial Dynamics in Neurodegenerative Diseases

The proper maintenance of mitochondrial dynamics is crucial to the proper function of the cell and the health of the organism. For example, as reviewed by Madan et al., loss of fission is thought to maintain the undifferentiated state of a cell, while an increase in fusion increases ATP potential and the polarization of hepatocytes [25]. Cells under starvation conditions will increase fusion and decrease fission to increase their ATP potential [24]. This is supported by the fact that diabetic and obese patient pancreatic cells have fragmented mitochondria [24]. Additionally, there is extensive remodeling of mitochondria during embryogenesis and mutations within fission and fusion proteins result in developmental defects, some of which are lethal [25]. Finally, it is clear that dynamics are altered to ensure that ROS levels are regulated. Excessive ROS induces recruitment of Drp1 to the mitochondria driving fission events. This decreases membrane potential and ATP production [24].

In terms of neurodegeneration, fission is required for autophagy (removal of damaged organelles) [37,38]. If mitochondrial dynamics are disrupted in such a way that autophagy cannot be carried out, then these cells not only have nonfunctional mitochondria, but they will also have an increase in ROS, a hallmark of neurodegenerative diseases [24].

On the other hand, mitochondrial fusion is used to repair mitochondria [37,38], thus loss of fusion has also been shown to cause neurodegeneration [103]. This alteration of fission and fusion has been noted in Alzheimer’s disease with an increase in fission and decrease in fusion [24,29,103]. An increase in fission events has also been noted in Parkinson’s disease [24,29,103] and Huntington’s disease [31], while a decrease in fusion has been noted in both Charcot Marie Tooth and Dominant Optic Atrophy [24]. The connection with motility is not as clear. Some Alzheimer’s disease patients show a hyperphosphorylation of the tau protein. This decreases the stability of the microtubules and thus decreases mitochondrial motility, and subsequently alters the balance of fission and fusion [103].

## 10. Conclusions

Currently, the role microtubules and actin play in mitochondrial dynamics is not fully understood in any model organism, and next to nothing is known about the function of these filaments in the alternative mitochondrial dynamics found in *D. discoideum*. Numerous questions remain, such as how do mitochondria move on their respective cytoskeletal tracks outside of mammalian models? Are motor proteins involved in all systems? In systems that clearly use motors and cytoskeletal dynamics, how does the cell decide which method is used? In mammals, how do microtubules regulate fission and fusion? In budding yeast, is actin used to regulate fusion? In fission yeast, do microtubules help regulate fusion? What about simple model systems like *D. discoideum*? In *D. discoideum*, we know that microtubules are more involved than actin but how do they regulate fission and fusion, what is the molecular mechanism? Finally, the big picture: How are motility, fission, and fusion interconnected? Does one process drive the other? What proteins mediate the communication between these processes?

The answers to these questions will not only provide us with a better understanding of the evolution of mitochondrial dynamics, but more importantly, they will help us to understand how the cytoskeleton regulates mitochondrial dynamics. This will provide valuable insight into mitochondrial disease and neurodegeneration [87,104]. In the brain, neuron function is dependent on the dynamics of the mitochondria [105]. Disruptions in dynamics lead to fragmented mitochondria that have transport defects [30]. This leads to poor distribution of mitochondria in neurons, and therefore, less energy production in some areas [30]. Increases in fission, loss of fusion, and fragmented mitochondria are side effects in several neurodegenerative diseases such as Parkinson’s, Alzheimer’s, and Huntington’s disease [29,30,31]. There is no known cure for these diseases, but new therapeutic strategies using drugs to alter mitochondrial fusion and fission activity are being considered [31,106]. Tactics for recovering microtubule-dependent motility and axonal transport have also been considered [107]. The future of these therapeutic studies is dependent on an in-depth understanding of the mechanisms of mitochondrial dynamics. Understanding the cytoskeleton’s role in dynamics in the model organism *D. discoideum* and additional insight into yeast and mammals will further our understanding of these mechanisms and could potentially uncover new therapeutic targets for these neurodegenerative diseases.

## Figures and Tables

**Table 1:** Summary of mammalian proteins involved in microtubule-regulated fission, fusion, and motility described in this review.

Protein	Function
Kinesin-1	Microtubule motor protein
Kinesin-3	Microtubule motor protein
Cytoplasmic dynein	Microtubule motor protein
TRAK1/TRAK2	Adaptors linking microtubule motors to mitochondria
Miro (Mitochondrial Rho GTPase)	Adaptor that connects some TRAK1/2 adaptors to the mitochondria
Syntabulin	Adaptor between microtubules and kinesin-1
KIFBP (Kinesin Binding Protein)	Kinesin-3 adaptor linking motor to microtubules
Mitofusins (Mfn1/2)	GTPase responsible for mitochondrial fusion
Mtus1 (Microtubule-associated tumor suppressor 1)	Interacts with mitofusins to mediate fusion
Drp1 (Dynamin related protein 1)	GTPase responsible for mitochondrial fission

**Table 2:** Summary of what is currently known about the cytoskeleton’s involvement in mitochondrial dynamics in mammals, fission yeast, and budding yeast.

Model	Motility	Fission	Fusion	Citations
Mammals	Microtubules used as tracks primarily, but actin is also sometimes used as tracks in neurons for short distances. Actin may also anchor mitochondria in place. Unclear if actin-based motility is based on motors or actin dynamics.	Microtubules also affect rate of fission, mechanism unclear. Actin directly involved in fission initiation at sites of ER-mitochondria contact. Actin polymerization via INF2 but not ring formation drives mitochondrial constriction and then recruits Drp1 to complete scission of the organelle.	Microtubules affect rate of fusion, mechanism unclear; Actin attachment to mitochondria prohibits fusion.	[44,45,46,50,54,55,56,61,67,68,69,70,71,72,73]
Fission yeast	Microtubules used as tracks, Microtubule dynamics move mitochondria. No actin-based motility.	Microtubules block Dnm1-mediated fission; unclear if actin is involved.	Currently unknown	[58,59,60,61,62,66]
Budding yeast	No microtubule-based motility. Actin used as tracks, dynamics are the predominant motility mechanism.	Actin likely plays a role as evidenced by pro-fission actin regulatory protein-Srv2.	Currently unknown	[65,74,75,76,77,78]
